# Inflammation-related plasma protein levels and association with adiposity measurements in young adults

**DOI:** 10.1038/s41598-021-90843-x

**Published:** 2021-05-31

**Authors:** Susanna Klevebro, Sophia Björkander, Sandra Ekström, Simon K. Merid, Olena Gruzieva, Anders Mälarstig, Åsa Johansson, Inger Kull, Anna Bergström, Erik Melén

**Affiliations:** 1 Department of Clinical Science and Education, Södersjukhuset, Karolinska Institutet, Sjukhusbacken 10, 118 83 Stockholm, Sweden; 2 Sachs’ Children and Youth Hospital, Södersjukhuset, Stockholm, Sweden; 3 Institute of Environmental Medicine, Karolinska Institutet, Stockholm, Sweden; 4 Centre for Occupational and Environmental Medicine, Region Stockholm, Stockholm, Sweden; 5 Department of Medical Epidemiology and Biostatistics, Karolinska Institutet, Stockholm, Sweden; 6 Department of Immunology, Genetics and Pathology, Science for Life Laboratory, Uppsala University, Uppsala, Sweden

**Keywords:** Biomarkers, Medical research, Risk factors

## Abstract

Obesity-related inflammation is associated with cardiovascular, metabolic, and pulmonary diseases. The aim of this study was to demonstrate associations between adiposity measurements and levels of inflammation-related plasma proteins in a population of young adults. Subjects from a population-based birth cohort with a mean age of 22.5 years were included in the study population (n = 2074). Protein levels were analyzed using the Olink Proseek Multiplex Inflammation panel. Percentage body fat (%BF) and visceral fat rating (VFR) measurements were collected using Tanita MC 780 body composition monitor. Linear regression of standardized values was used to investigate associations. Potential effect modifications by sex and BMI category were assessed. Of 71 investigated proteins, 54 were significantly associated with all adiposity measurements [%BF, body mass index (BMI), VFR and waist circumference]. Among proteins associated with %BF, seven showed a larger or unique association in overweight/obese subjects and three showed a significant effect modification by sex. Fourteen proteins more strongly associated with VFR in females compared to males. Adipose-associated systemic inflammation was observed in this young adult population. Sex and adiposity localization influenced some of the associations. Our results highlight specific proteins as suitable biomarkers related to adiposity.

## Introduction

Obesity has been demonstrated to increase the risk of cardiovascular, metabolic and pulmonary disease^1, 2^, and chronic inflammation is believed to drive disease development^3, 4^. Adipose tissue is a complex and highly active metabolic endocrine organ. A variety of immune cells infiltrate and become resident in adipose tissue^5^, where they, along with adipocytes, secrete inflammatory factors^6^. Adipose tissue expansion induces an innate and adaptive immune response, and affect glucose metabolism and inflammation^7^. Visceral adipose tissue is wrapped around major abdominal organs and is an independent risk factor for cardiovascular and metabolic disease^8^. Visceral and subcutaneous adipose tissues differ in composition of infiltrated cells, and in their production of adipose-derived secreted factors^9–11^. Associations between adipose tissue and selected pro-inflammatory factors have been demonstrated in children, adolescents and adults^12–14^. Weight change and body mass index (BMI) have also been associated with several inflammation-related proteins in studies utilizing proteomic methods in cohorts of overweight and obese participants^15–17^.

Fat deposition differs between females and males. At comparable BMI, females have a higher percentage body fat while males have more lean mass. The fat more likely accumulate around hips and thighs in females and around the trunk and abdomen in males^18^. Animal models indicate that sex hormones, for example the estrogen to androgen ratio, influence adipose tissue deposition^19^. Sex differences in CRP levels have been correlated to differences in visceral- and subcutaneous adipose tissue^20^. Further, there are immunological differences between the sexes, as females mount stronger acute inflammatory responses to infectious agents and vaccines, but are also more vulnerable to chronic inflammatory conditions^21^. How adiposity measurements associate with a large panel of inflammation-related biomarkers in a young population including both normal and overweight subjects has not been previously studied.

The primary aim of this study was to demonstrate associations between adiposity measurements and levels of inflammation-related plasma proteins in a population of normal- and overweight/obese young adults. The secondary aim was to assess interaction between body fat and sex regarding association with inflammation-related protein levels.

## Results

### Characteristics of the study population

**Figure 1:**
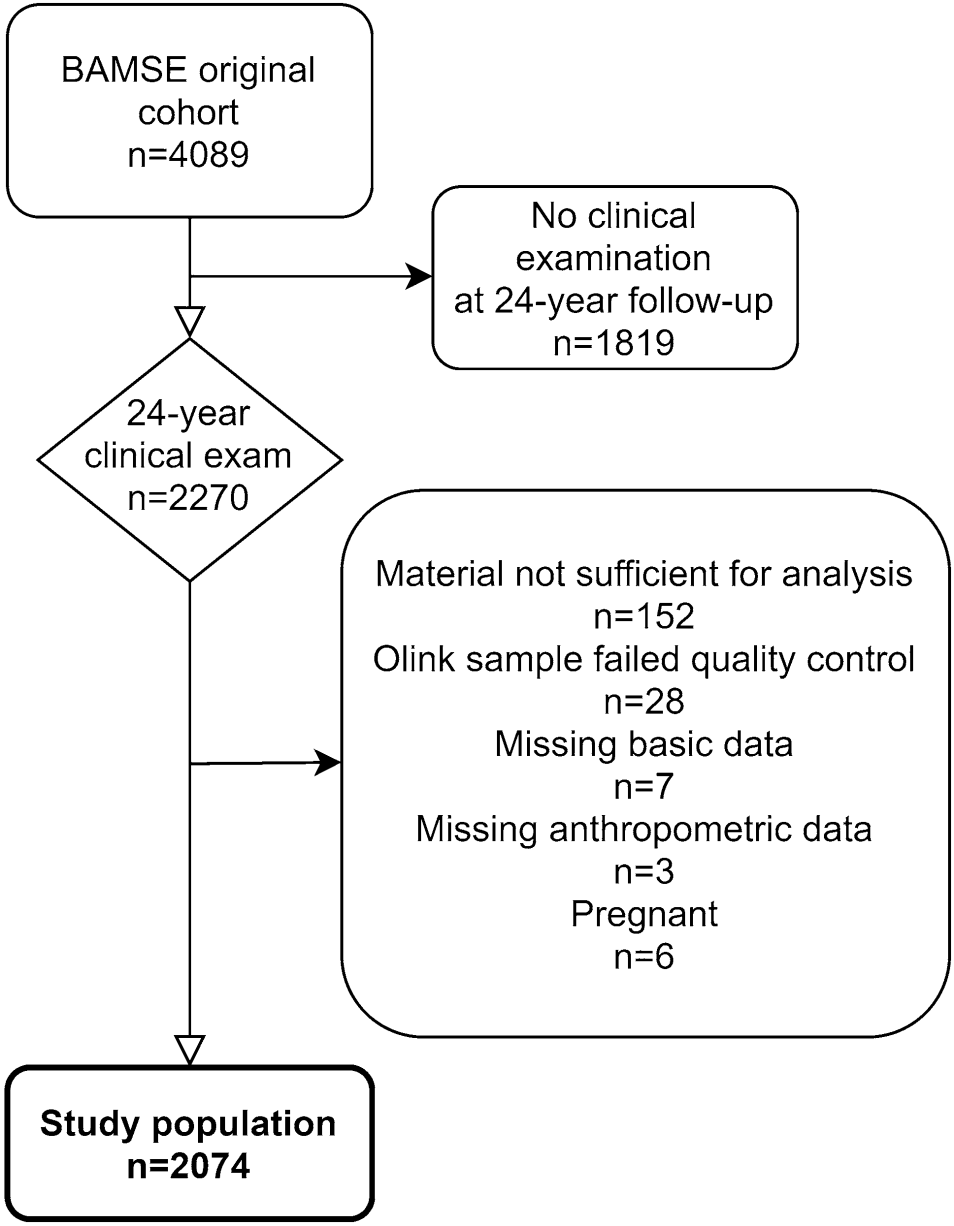
Flow chart of the study population from the BAMSE cohort.

In total, 2074 subjects (1147 females and 927 males) were included in the final study population (Fig. 1). In comparison with subjects from the original population-based cohort, the final study population showed a higher proportion of females (Supplementary Table S1). As expected, males and females differed in their anthropometric measurements (Table 1). Males had higher BMI but lower body fat % and reported a higher level of physical activity compared to females. More females were smokers and more males used e-cigarettes and snuff. Protein levels differed between the sexes for 54 of the 71 proteins (i.e. nominal p-value < 0.05 for association). For most of the proteins, the association between sex and protein level was not related to differences in body composition, as demonstrated by including %BF, BMI and VFR as covariates in separate regression models (Supplementary Table S2). Median values were lower in females compared to males for 47 of the proteins. Mean, standard deviation, median and 25th–75th percentile of NPX values are demonstrated in Supplementary Table S2.

**Table 1:** Descriptive characteristics of the study population (n = 2074).

Variable	Females (n = 1147)			Males (n = 927)			p-value*
	n	Mean (sd)	Median (p25–p75)	n	Mean (sd)	Median (p25–p75)	
Age (y)	1147	22.6 (0.6)	22.5 (22.2–22.9)	927	22.7 (0.6)	22.6 (22.2–22.9)	0.320
Weight (kg)	1147	64.5 (11.2)	62.7 (57.1–69.8)	927	78.1 (13.5)	76.6 (69.1–85.2)	< 0.001
Height (m)	1147	1.68 (0.06)	1.68 (1.64–1.72)	927	1.82 (0.08)	1.82 (1.78–1.87)	< 0.001
BMI (kg/m^2^)	1147	22.8 (3.7)	22.1 (20.3–24.1)	927	23.5 (3.7)	22.9 (21.1–25.1)	< 0.001
Body fat (%)	1147	26.5 (6.2)	26.1 (22.4–30.4)	927	16.8 (6.1)	15.8 (12.5–20.1)	< 0.001
VFR (score)	1147	2 (1.5)	1 (1–2)	926	3 (2.9)	2 (1–4)	< 0.001
Waist circ (cm)	1141	75 (9)	73 (69–80)	923	84 (10)	83 (78–89)	< 0.001
**Smoking habits**	1146			924			0.014
Do not smoke		n = 742	64.8%		n = 645	69.8%	
Used to smoke		n = 140	12.2%		n = 117	12.7%	
Sometimes		n = 163	14.2%		n = 108	11.7%	
Every day		n = 101	8.8%		n = 54	5.8%	
E cigarette use	1145	n = 29	2.5%	924	n = 48	5.2%	0.001
Snuff use	1146	n = 75	6.5%	925	n = 207	22.4%	< 0.001
**PA level**	948			783			0.001
Low		n = 159	16.8%		n = 102	13.0%	
Moderate		n = 294	31.0%		n = 181	23.1%	
High		n = 495	52.2%		n = 500	63.9%	
**BMI category at 24**	1147			927			0.007
Underweight		n = 70	6.1%		n = 51	5.5%	
Normal weight		n = 854	74.5%		n = 637	68.7%	
Overweight		n = 175	15.3%		n = 191	20.6%	
Obesity		n = 48	4.2%		n = 48	5.2%	

*Mann–Whithney U-test or Chi^2^.

### Adiposity measurements and inflammation-related proteins

**Figure 2:**
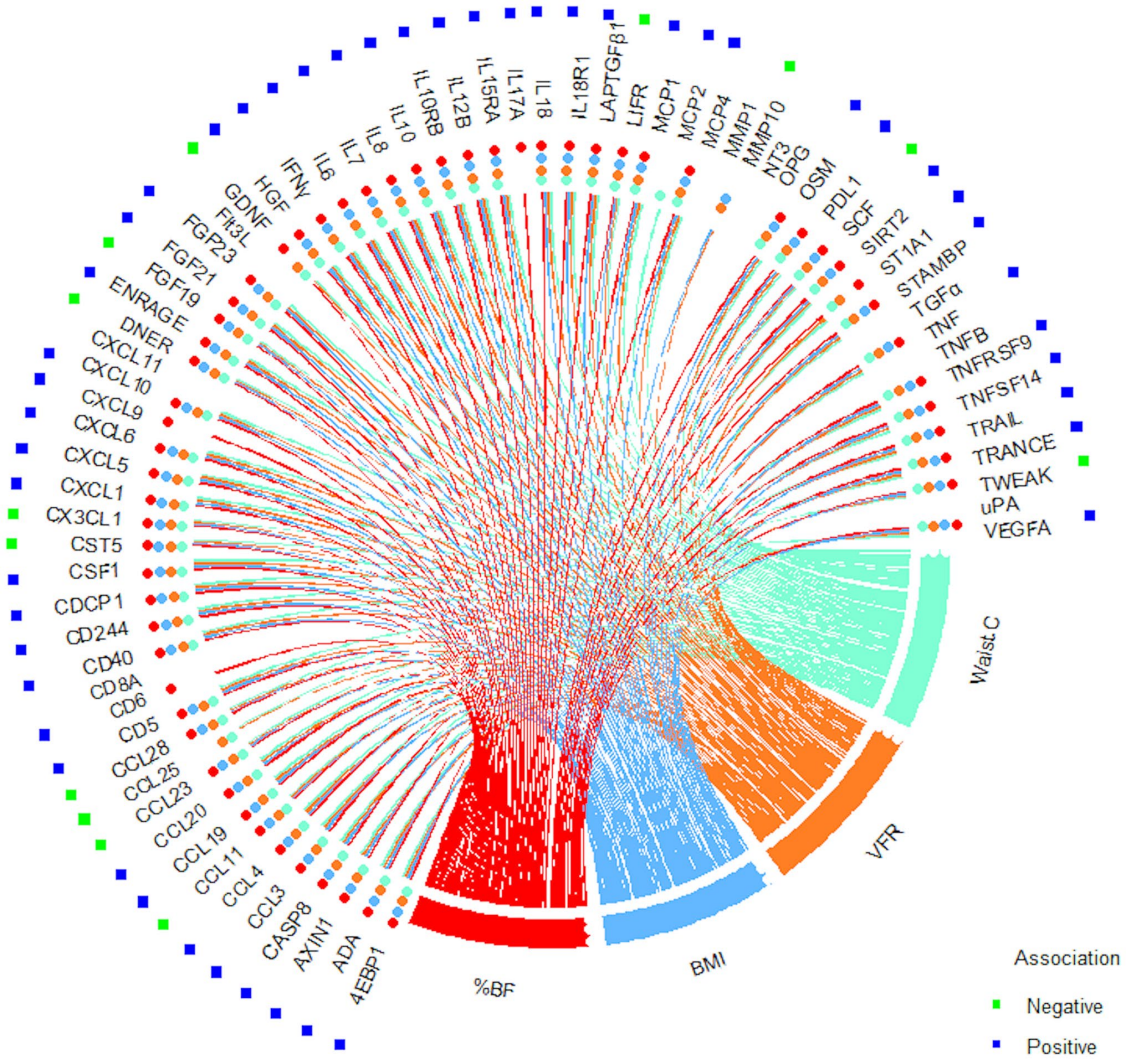
Associations between body composition measurements and protein levels. Statistically significant (i.e. nominal p-value < 0.05 at < 0.05 FDR) negative (green dot) and positive (blue dot) association with %BF (red line and dot), BMI (light blue line and dot), VFR (orange line and dot), and waist circumference (light green line and dot) are shown in circos plot based on results from linear regression models of %BF, BMI, VFR and waist circumference respectively. Adjusted for sex, smoking, e-cigarette use, snuff use, and age at sampling.

All adiposity measurements in this study were associated with the level of most of the inflammation-related plasma proteins in the Olink panel. Figure 2 demonstrates positive and negative associations with %BF, BMI, VFR, and waist circumference based on the results from regression analyses adjusted for sex, smoking, e-cigarette use, snuff use, and age at follow-up (complete results presented in Supplementary Table S3). Of the 71 proteins, 54 were associated with all four adiposity measurements and most associations were in a positive direction of effect. In our study population, association with %BF was apparent for 58 of the proteins at an FDR of 5%.

### Effect modification by sex on associations between %BF and inflammation-related proteins

**Figure 3:**
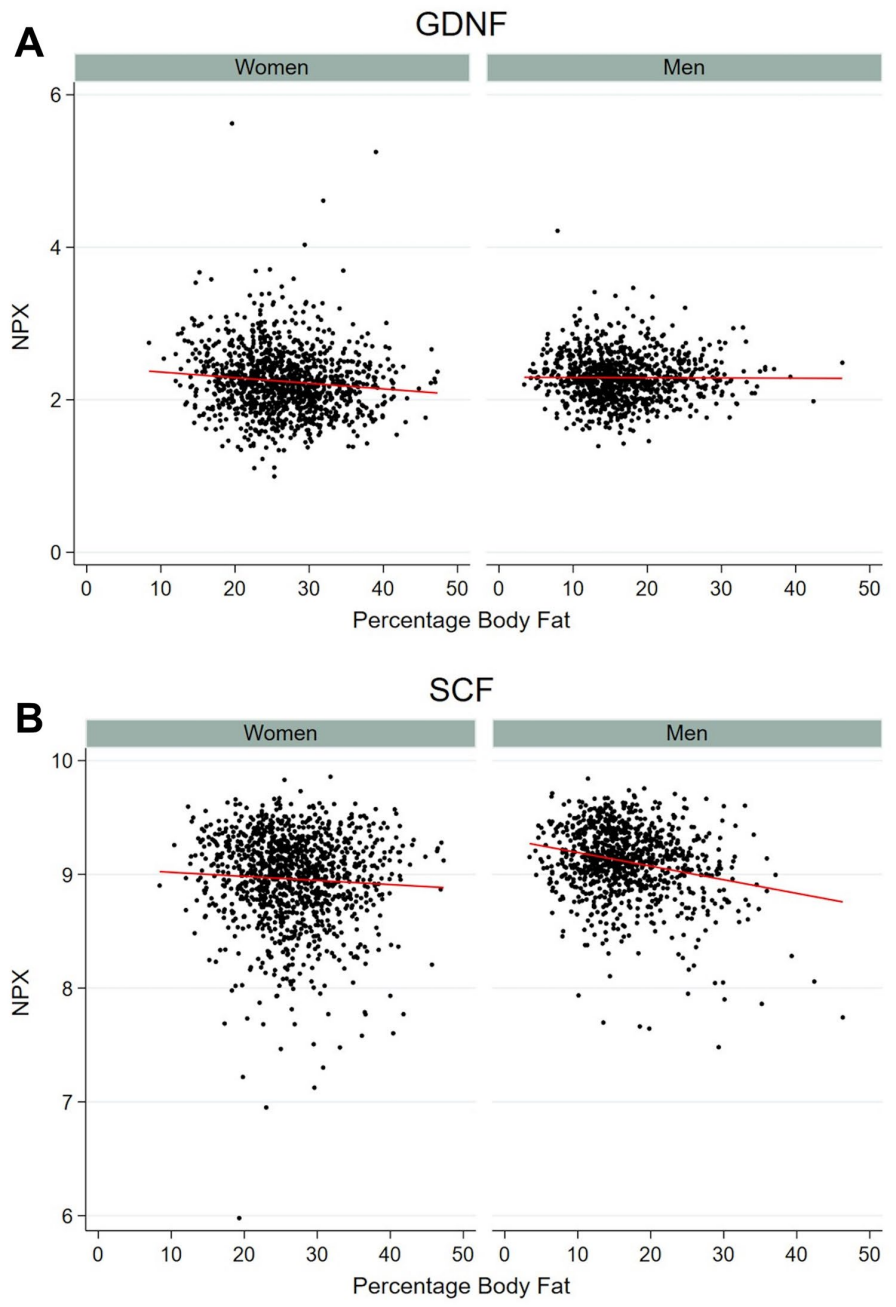
Examples of effect modification by sex. GDNF (**A**) and SCF (**B**) and association with %BF in females and males. Scatterplot and regression line of protein levels (in NPX) and %BF in females and males respectively.

Three of the 58 proteins associated with %BF demonstrated effect modification by sex at an FDR of 5% (Table 2). Glial cell line-derived neurotrophic factor (GDNF) was negatively associated with %BF in females but not in males, whereas Stem Cell Factor (SCF) had a strong negative association with %BF in males but not in females. These examples are illustrated in scatterplots of raw data with regression lines of the association in females and males respectively in Fig. 3. Interleukin-18 receptor 1 (IL18R1) had a stronger positive association with %BF in males compared to females. The effect modification was not related to differences in BMI between the sexes. All six proteins shown in Table 2 had a p-value for the interaction term < 0.05 also in a model with BMI included as a covariate (data not shown).

**Table 2:** Association between %BF and protein levels in females and males for proteins with effect modification by sex.

Protein	Females			Males			p-value interact	p-value interact*
	Coef	95% CI	p-value	Coef	95% CI	p-value		
CDCP1	0.43	0.33 to 0.53	< 0.001	0.58	0.48 to 0.67	< 0.001	0.264	0.027
GDNF	− 0.21	− 0.31 to − 0.11	< 0.001	0.00	− 0.08 to 0.08	0.947	0.047	0.001
IL10	0.05	− 0.04 to 0.15	0.240	0.20	0.10 to 0.30	< 0.001	0.264	0.024
IL18R1	0.41	0.31 to 0.51	< 0.001	0.60	0.51 to 0.69	< 0.001	0.048	0.002
IL6	0.67	0.59 to 0.74	< 0.001	0.52	0.43 to 0.61	< 0.001	0.264	0.025
SCF	− 0.09	− 0.19 to 0.00	0.064	− 0.31	− 0.41 to − 0.21	< 0.001	0.048	0.002

Stratified analyses in men and women from linear regression of transformed protein levels adjusted for smoking, e-cigarette use, snuff use, and age at follow-up.

The table includes all proteins with unadjusted p-value for the interaction term %BF#Sex of < 0.05.

p-values at 0.05 FDR.

*p-value for the interaction term without FDR correction.

### Effect modification by BMI on associations between %BF and inflammation-related proteins

**Figure 4:**
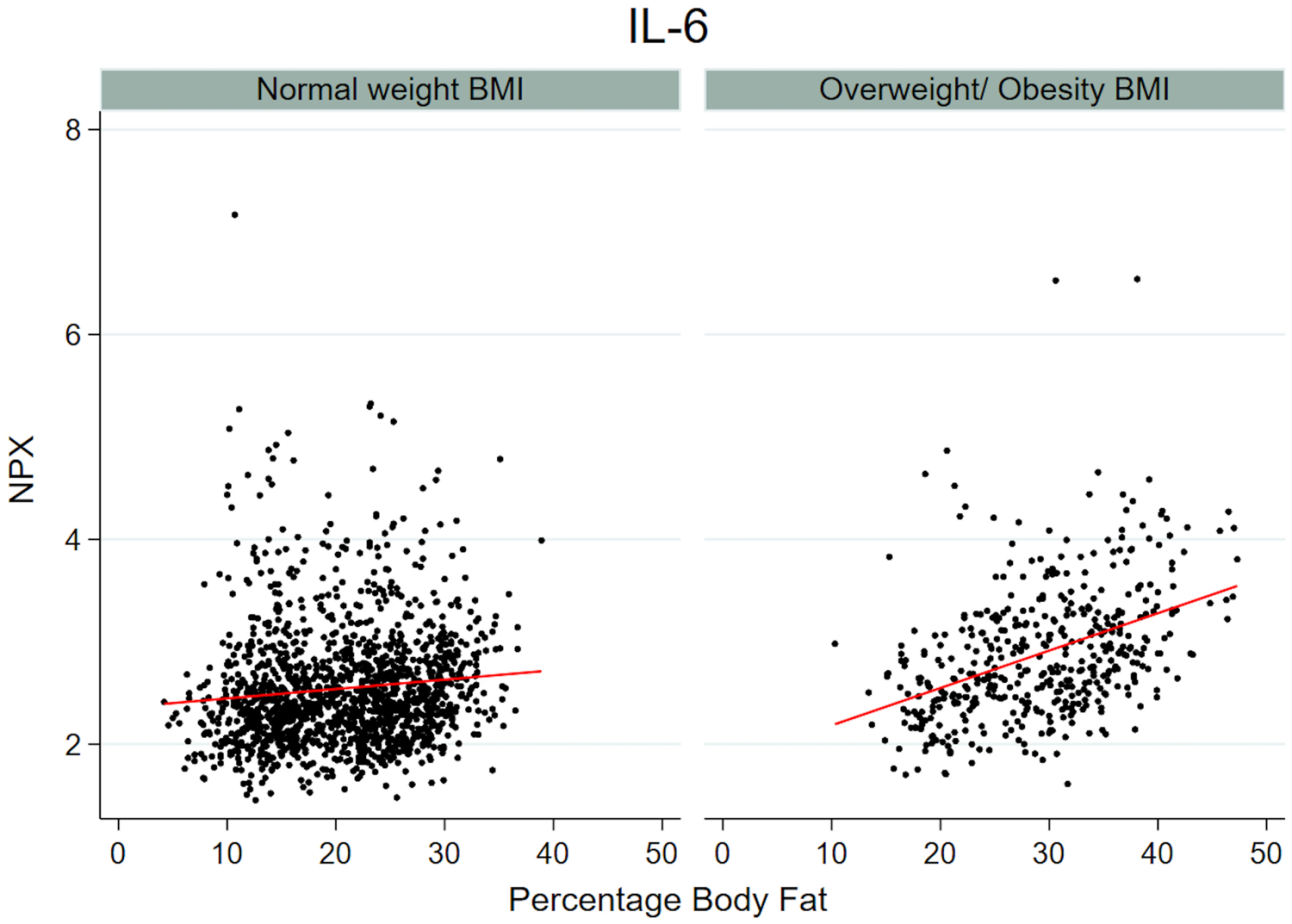
Example of effect modification by BMI category. IL-6 and association with %BF in normal weight and overweight/obese subjects. Scatterplot and regression line of protein level (in NPX) and %BF in normal weight and overweight/obese BMI groups respectively.

To examine if the association between %BF and inflammation-related proteins differed in normal weight versus overweight/obese subjects (BMI ≥ 25) an interaction term between BMI category and %BF was introduced in the model. Seven proteins (CDCP1, FGF23, HGF, IL-6, LAPTGFβ1, MCP-1, and MCP-4) had significant effect modification of BMI category, showing a stronger or unique association with %BF in the overweight/obese group compared to the normal weight group (Table 3). For example, Interleukin-6 (IL-6) was positively associated with %BF in both groups, although the effect was larger in the overweight/obese group (Fig. 4). Due to small numbers, analyses stratified in three groups of normal weight, overweight and obese subjects resulted in large confidence intervals. It was not possible to show statistically significant differences in association with %BF between overweight and obese subjects. CDCP1, FGF23, HGF and IL-6 still had more prominent positive associations with %BF in overweight compared to normal weight subjects and the coefficients did not indicate that the associations were driven by obesity, whereas MCP-1 had a clear positive association with %BF only in the obese group (Supplementary Table S4).

**Table 3:** Association between %BF and protein levels stratified by BMI categories.

Protein	Normal weight (18.5 to < 25 kg/m^2^)			Overweight/ obesity (≥ 25 kg/m^2^)			p-value interact	p-value interact*
	Coef	95% CI	p-value	Coef	95% CI	p-value		
CCL3	0.34	0.23 to 0.46	< 0.001	0.60	0.43 to 0.77	< 0.001	0.159	0.041
CCL20	0.09	− 0.03 to 0.20	0.232	0.37	0.19 to 0.55	< 0.001	0.120	0.023
CDCP1	0.27	0.16 to 0.38	< 0.001	0.88	0.71 to 1.05	< 0.001	0.005	< 0.001
DNER	− 0.19	− 0.31 to − 0.08	0.007	− 0.30	− 0.47 to − 0.12	0.002	0.161	0.044
FGF21	0.30	0.19 to 0.42	< 0.001	0.74	0.58 to 0.91	< 0.001	0.111	0.019
FGF23	0.07	− 0.05 to 0.19	0.343	0.56	0.39 to 0.72	< 0.001	0.030	0.003
HGF	0.12	0.01 to 0.23	0.082	0.72	0.57 to 0.87	< 0.001	< 0.001	< 0.001
IL6	0.50	0.39 to 0.61	< 0.001	0.86	0.72 to 1.00	< 0.001	< 0.001	< 0.001
IL18	0.14	0.02 to 0.26	0.057	0.46	0.30 to 0.63	< 0.001	0.157	0.035
LAPTGFbeta1	− 0.03	− 0.14 to 0.09	0.733	0.33	0.15 to 0.50	0.001	0.032	0.004
MCP1	− 0.10	− 0.21 to 0.01	0.153	0.36	0.20 to 0.53	< 0.001	0.030	0.003
MCP4	− 0.08	− 0.20 to 0.04	0.255	0.27	0.11 to 0.44	0.003	0.005	< 0.001
OSM	0.02	− 0.10 to 0.13	0.843	0.35	0.18 to 0.52	< 0.001	0.111	0.018
TNFSF14	0.15	0.04 to 0.26	0.034	0.38	0.21 to 0.54	< 0.001	0.121	0.025

Stratified analyses in BMI categories from linear regression of transformed protein levels adjusted for sex, smoking, e-cigarette use, snuff use, and age at follow-up.

The table includes all proteins with unadjusted p-value for the interaction term %BF#BMI_category of < 0.05.

p-values at 0.05 FDR.

*p-value for the interaction term without FDR correction.

### Physical activity and association between %BF and inflammation-related proteins

Of the 2074 participants, 1728 had information regarding physical activity. Including physical activity as a covariate did not alter the main results, Supplementary Table S5. Effect modification of physical activity (as a binary variable of low/moderate vs high) was examined by including an interaction term in the adjusted model of %BF. At a 5% FDR the interaction term was not significant for any of the proteins.

### VFR and inflammation-related proteins

Of the 71 proteins analyzed, 55 were associated with VFR in our study population, Supplementary Table S3. Sex modified the association between VFR and protein level in 15 of the 55 proteins, Table 4. The effect was larger in females compared to males for 14 of the proteins. As with %BF, SCF demonstrated a negative association with VFR only in males. GDNF, that differed in association with %BF between females and males, was not associated with VFR at all. The proteins that were modified by BMI category in the association with %BF did not demonstrate differences in association with VFR at a 5% FDR of the interaction term. Leukemia inhibitory factor receptor (LIFR) was negatively associated with all adiposity measurements, Fig. 2, Supplementary Table S3, and showed a significant negative association with VFR only in the normal weight BMI group, Table 5.

**Table 4:** Association between visceral fat rating and protein levels in females and males.

Protein	Females			Males			p-value interact	p-value interact*
	Coef	95% CI	p-value	Coef	95%	p-value		
4EBP1	0.08	0.05 to 0.12	< 0.001	0.04	0.02 to 0.06	< 0.001	0.113	0.040
ADA	0.09	0.06 to 0.13	< 0.001	0.03	0.01 to 0.04	0.003	0.008	0.001
AXIN1	0.09	0.05 to 0.13	< 0.001	0.04	0.02 to 0.06	< 0.001	0.098	0.034
CCL3	0.18	0.14 to 0.21	< 0.001	0.09	0.07 to 0.11	< 0.001	0.002	< 0.001
CCL19	0.12	0.08 to 0.16	< 0.001	0.05	0.03 to 0.07	< 0.001	0.008	0.001
CCL28	− 0.09	− 0.13 to − 0.05	< 0.001	− 0.03	− 0.05 to − 0.01	0.007	0.017	0.004
CD5	0.09	0.05 to 0.12	< 0.001	0.03	0.01 to 0.05	0.004	0.013	0.003
CDCP1	0.20	0.17 to 0.24	< 0.001	0.13	0.11 to 0.15	< 0.001	0.007	0.001
DNER	− 0.07	− 0.11 to − 0.03	< 0.001	− 0.03	− 0.05 to − 0.01	0.015	0.098	0.034
HGF	0.16	0.13 to 0.20	< 0.001	0.09	0.07 to 0.10	< 0.001	0.002	< 0.001
IL6	0.26	0.22 to 0.29	< 0.001	0.11	0.08 to 0.13	< 0.001	< 0.001	< 0.001
IL12B	0.11	0.07 to 0.14	< 0.001	0.04	0.01 to 0.06	0.002	0.013	0.002
IL18	0.12	0.09 to 0.15	< 0.001	0.07	0.04 to 0.09	< 0.001	0.029	0.007
MCP1	0.11	0.08 to 0.15	< 0.001	0.03	0.01 to 0.05	0.001	0.002	< 0.001
MCP4	0.09	0.05 to 0.13	< 0.001	0.03	0.01 to 0.05	0.002	0.037	0.010
SCF	− 0.01	− 0.05 to 0.03	0.544	− 0.08	− 0.10 to − 0.06	< 0.001	0.013	0.002
TNFRSF9	0.06	0.02 to 0.10	0.004	0.01	0.00 to 0.03	0.153	0.098	0.031
TNFSF14	0.13	0.09 to 0.16	< 0.001	0.06	0.04 to 0.08	< 0.001	0.008	0.001
TRAIL	0.11	0.07 to 0.14	< 0.001	0.06	0.04 to 0.08	< 0.001	0.052	0.015
VEGFA	0.15	0.12 to 0.19	< 0.001	0.08	0.05 to 0.11	< 0.001	0.013	0.003

Stratified analyses in men and women from linear regression of transformed protein levels adjusted for smoking, e-cigarette use, snuff use, and age at follow-up.

The table includes all proteins with unadjusted p-value for the interaction term %BF#sex of < 0.05.

p-values at 0.05 FDR.

*p-value for the interaction term without FDR correction.

**Table 5:** Association between visceral fat rating and protein levels stratified by BMI categories.

Protein	Normal weight (18.5 to < 25 kg/m^2^)			Overweight/obesity (≥ 25 kg/m^2^)			p-value interact	p-value interact*
	Coef	95% CI	p-value	Coef	95% CI	p-value		
CCL3	0.15	0.10 to 0.21	< 0.001	0.10	0.07 to 0.12	< 0.001	0.201	0.018
CCL11	− 0.12	− 0.17 to − 0.07	< 0.001	− 0.03	− 0.05 to 0.00	0.057	0.020	0.001
CCL23	− 0.04	− 0.10 to 0.02	0.331	0.03	0.01 to 0.06	0.010	0.208	0.023
IL6	0.18	0.12 to 0.24	< 0.001	0.13	0.09 to 0.17	< 0.001	0.068	0.005
LIFR	− 0.16	− 0.21 to − 0.10	< 0.001	− 0.02	− 0.05 to 0.00	0.113	< 0.001	< 0.001
MCP1	− 0.04	− 0.09 to 0.01	0.299	0.06	0.04 to 0.09	< 0.001	0.045	0.002
SCF	− 0.03	− 0.09 to 0.02	0.366	− 0.06	− 0.09 to − 0.03	< 0.001	0.305	0.039

Stratified analyses in BMI categories from linear regression of transformed protein levels adjusted for sex, smoking, e-cigarette use, snuff use, and age at follow-up.

The table includes all proteins with unadjusted p-value for the interaction term VFR#BMI_category of < 0.05.

p-values at 0.05 FDR.

*p-value for the interaction term without FDR correction.

## Discussion

In our population-based study of young adults, we show that adiposity reflects in a systemic inflammatory protein profile. Some of the proteins demonstrated a more marked positive association with %BF in overweight/obese subjects, suggestive of an accelerated release of these factors with increasing BMI, possibly promoting a chronic inflammatory state that has previously been related to increased risk of cardiometabolic disease development^3^. For most proteins, the association between protein level and total %BF was similar between the sexes. Observed differences in protein levels between females and males may be related to biological differences but also partly to differences in adiposity. Our results show that some protein levels associated differently to adiposity depending on sex. For example, there was a more prominent positive association with VFR in females compared to males for several proteins, indicating that the relation of visceral fat and the inflammatory profile differs between females and males.

The positive association between inflammatory protein levels and %BF in this study could indicate a spill-over of a local inflammation or be the result of a feed-back loop of increasing inflammation, in the adipose tissue. The effects of increased circulating inflammatory protein levels are likely to have systemic effects and implications for health. Obesity in childhood is likely to persist into adulthood^22^, and elevated levels of inflammatory factors in childhood and adolescence have been shown to track into adulthood^23^. Even moderate overweight has been associated with an increased risk of cardiovascular disease^24^. Increased risk of airway obstruction with increasing BMI has previously been demonstrated in this cohort^25^. The same study also demonstrated effect modification by sex in some of the associations. Longitudinal, large scale analysis of plasma proteins, that have been performed in predominantly select groups of patients, have shown that inflammatory patterns are influenced by weight loss and weight maintenance^15–17^, indicating potential benefit of a weight loss intervention. Similar patterns related to body fat and distribution have also been demonstrated in a population based study of elderly^26^. Physical activity has been shown to influence weight and inflammatory biomarkers^27^. In our cross-sectional study however, we found no significant interaction between %BF and physical activity in the association with protein levels. Dietary interventions might also be of importance and for example plant based diets have been associated with reduced levels of inflammatory biomarkers such as IL-6^28^.

IL-6 is one of the major pro-inflammatory factors released from adipose tissue and higher levels have also previously been associated with obesity as well as diseases like diabetes and asthma^29–31^. However, IL-6 plays highly complex role in metabolic regulation and can be secreted from adipocytes, adipose tissue macrophages and other adipose cell types^32^. Adipocyte-derived IL-6 was shown to accumulate adipose tissue macrophages without influencing glucose or insulin tolerance, while myeloid-cell derived IL-6 suppressed the polarization of M1 macrophages and improved tolerance^33^. In the current study, IL-6 was positively associated with %BF in both females and males and accelerated in overweight/obese subjects. The positive association of IL-6 and %BF did not differ significantly between females and males; however, IL-6 showed a stronger association with VFR in females. Several other proteins also demonstrated stronger positive association with VFR in females compared to males. Females have a higher %BF and a lower visceral fat mass compared to males and sex hormones are involved in fat mass regulation and distribution^34^. A previous study showed a larger effect of visceral fat on the risk of cardiovascular diseases and type 2 diabetes in females compared to males^35^. The results from our study support the theory that the association between visceral fat and inflammation may differ depending on sex.

Obesity-related risk of metabolic disease is affected by age, sex, total body fat content, and body fat distribution^36^. Especially an excess amount of visceral fat associates with metabolic syndrome and cardiovascular disease^8, 34, 35, 37^. Abundance and function of adipocytes and macrophages differ depending on fat mass localization^6^. Macrophages are the most abundant immune cell in adipose tissue and can constitute 50% of immune cells in obese conditions, to be compared with 10% in lean adipose tissue^38^. MCPs (Monocyte chemotactic proteins) are key factors in the regulation of monocyte/macrophage migration and infiltration. Higher levels of MCP have been associated with obesity and adipose tissue localization^10, 39, 40^. MCP-1 and MCP-4 showed significant positive correlation with %BF only in overweight/obese subjects in our study. Further, MCP-1 was positively associated with VFR in this group.

Several proteins demonstrated significant association with %BF in the normal weight population, with similar effect estimates in overweight/obese subjects. These proteins might be less relevant as indicators of adiposity-related inflammation and might reflect a general increase in body size. A few proteins were negatively associated with %BF in this study. SCF was negatively associated with %BF in males, and GDNF in females. SCF promotes brown adipocyte differentiation, contributes to mitochondrial function and energy expenditure^41, 42^ and low levels of SCF has been associated with increased incidence of cardiovascular events^43^. The browning capacity of adipose tissue and differentiation of precursor cells to beige adipocytes have been associated with metabolic conditions^44^. GDNF is involved in neuron survival and regeneration and has been described in several inflammatory conditions. Studies in rodents have demonstrated a protective effect against obesity of GDNF^45, 46^. All adiposity measurement had a negative association with LIFR. LIF, one of the ligands for LIFR, influence adipocyte differentiation^47, 48^. Hence, we speculate that the LIF-LIFR signaling might be impaired in obesity. Indeed, a negative association between LIFR and VFR was not observed in the overweight/obese group.

A limitation to this study was that the plasma protein levels we have measured reflect the combined proteome from many cell types and tissues. We are not able to trace the origin of the proteins and differentiate proteins secreted by macrophages, adipocytes, or other cells. Also, tissue levels and tissue effects are unknown. All the factors measured in this study are “inflammation-related” in the sense that they may be up- or downregulated during infection/inflammation, still their presence may not be a sign of chronic inflammation. The participants in this study were clinically healthy at the time of the study visit but we were not able to objectively rule out presence of inflammatory conditions. Another limitation is that protein expression was measured only on one occasion, limiting the inference of dynamic relationships between body composition and systemic inflammation.

There are several methods that measure adiposity and body composition. In this study we used a combination of bioimpedance and anthropometric measurements. Anthropometric measurements are easy to use in large cohort studies. Bioimpedance gives additional objective information regarding actual adiposity and fat mass location. Bioimpedance is a non-invasive, relatively cheap method and measurements of %BF correlates well with dual‐energy X‐ray absorptiometry^49^. The measurement of visceral fat by bioimpedance is not as accurate as measurement by MRI or DT^50, 51^, which are not feasible methods in cohorts of this size.

Our results show that adiposity is associated with the levels of inflammation-related markers in a young adult population with a normal distribution of BMI. Overweight/obesity strongly correlate with the levels of specific inflammatory markers, including IL-6. We also demonstrate that sex and adiposity localization influence these associations. The results highlight differences of importance when using inflammation-related plasma proteins as biomarkers associated with adiposity. Our study show that adiposity-driven inflammation can be observed in young adults before potential development of obesity-related diseases. The findings might have implications for targeted interventions aiming to reduce the inflammatory load in early adulthood.

## Methods

### Study design and study population

The study population was based on participants in the BAMSE (Swedish abbreviation for Child (Barn), Allergy, Milieu, Stockholm, Epidemiological) cohort, a Swedish population-based cohort of 4089 children born in Stockholm 1994–1996^52^. The children have been followed through repeated administration of questionnaires and have been invited to undergo clinical examinations at ages 4, 8, 16 and 24 years. Participants of the clinical examination at the 24-year follow-up, who had complete data regarding biomarkers and bioimpedance measurements, were included in the present study. Pregnancy was the only exclusion criterium. In total, 2270 subjects participated in the clinical examination and 2074 subjects with a median age of 22.5 years (range 20.9–25.2 years) were included in the final study population (Fig. 1).

### Clinical investigation

Venous blood was collected in EDTA tubes (BD Vacutainer^®^) at the 24-year follow-up. Fasting prior to sampling was not required. Participants were asked to re-schedule the follow-up visit if not feeling well but no test to evaluate presence of acute inflammation was performed. Plasma was obtained by centrifugation, aliquoted and stored at – 80 °C until analyzed. Height was measured twice to the nearest 0.5 cm using a wall-mounted stadiometer, and the mean value was used for analyses. Waist circumference was measured at the end of an expiration below elbow level. Weight and bioimpedance measurements were taken using Tanita MC 780 body composition monitor according to instructions from the manufacturer. In the present study, we included body mass index (BMI), percentage body fat (%BF) and visceral fat rating (VFR) measurements. The measurement of visceral fat was expressed as a rating from 1 to 60 developed by the manufacturing company^50, 51^. BMI was used as a continuous variable in analyses of association with protein level. In stratified analyses, BMI was categorized in two categories, normal weight (18.5 to < 25 kg/m^2^) and overweight including obesity (≥ 25 kg/m^2^). Level of physical activity was defined based on time spent on moderate and vigorous intensity activities reported in the 24-year questionnaire. The answers were categorized according to IPAQ^53^ as high (≥ 7 h per week of moderate to vigorous activity or ≥ 3.5 h per week of vigorous activity), moderate (≥ 2.5 h per week of moderate to vigorous activity) or low (< 2.5 h per week of moderate to vigorous activity) level of physical activity. Smoking was categorized into daily smoking, occasional smoking, and no current smoking.

### Proseek multiplex inflammation panel

The expression of 92 inflammation-related protein biomarkers in plasma were analyzed by the Proseek Multiplex Inflammation Panel (version 95302) from Olink Biosciences, Uppsala, Sweden. Assay characteristics and validations are available from the manufacturer’s webpage (https://www.olink.com/resources-support/document-download-center/#). In brief, antibodies labelled with complementary oligonucleotide sequences were allowed to bind pairwise to the target protein. Upon DNA-polymerization, the paired oligonucleotides form a reporter sequence that was amplified by qRT-PCR. Data are expressed as Normalized Protein Expression (NPX) units on a log2 scale calculated from normalized Ct values. Samples that deviated more than 0.3 NPX from the median value of an internal control were excluded. The lower limit of detection (LOD) was defined as three standard deviations above background. 71 proteins with > 75% of samples above LOD were included in the analyses and, in accordance with recommendations by the company, values below LOD were not replaced by arbitrary values. The full names of the proteins are given in Supplementary Table S6.

### Statistical methods

All statistical analyses were performed using Stata version 16 (StataCorp LP, College Station, TX, USA). Circos plots were constructed using the circlize package in R version 3.6.1 (R Foundation for Statistical Computing, Vienna, Austria). Median, 25th, and 75th percentiles are presented for continuous variables, number and percentage for categorical variables, and comparison between groups were tested using Mann–Whitney U-test or Chi-2. Linear regression with robust standard errors was used to investigate associations between adiposity measurements and protein levels. Protein values were standardized using rank-based inverse normal transformation. Significance was based on a false discovery rate (FDR) of 5% using the Benjamini–Hochberg procedure^54^. Based on principal component regression analysis, covariates considered as potential confounders included sex, smoking, e-cigarette use, snuff use, age at follow-up, and level of physical activity. Information regarding physical activity was not available from all study subjects and therefore not included in the main regression model. A sensitivity analysis of the primary outcome that included the level of physical activity was performed. Potential effect modifications by sex as well as BMI category were examined by introducing an interaction term in the regression model. Effect modification was considered significant based on a 5% FDR. Stratified results are presented for all nominally significant associations.

### Ethics statement

The study was conducted in accordance with the Declaration of Helsinki and approved by the Regional Ethics Committee in Stockholm (DNR 2016/1380-31/2). All participants in this study were over the age of 18 years and provided written informed consent. At previous follow-ups of the BAMSE study, informed consent has also been collected from a parent or legal guardian.

## Supplementary Information

Supplementary Tables.

## Abbreviations

%BF: Percentage body fat
BMI: Body mass index
CDCP: CUB domain-containing protein
FDR: False discovery rate
FGF: Fibroblast growth factor
GDNF: Glial cell line-derived neurotrophic factor
HGF: Hepatocyte growth factor
IL: Interleukin
LAPTGF: Latency-associated peptide transforming growth factor
LIFR: Leukemia inhibitory factor receptor
LOD: Limit of detection
MCP: Monocyte chemotactic protein
NPX: Normalized protein expression
SCF: Stem cell factor
VFR: Visceral fat rating
