# Supplementary material for: Inflammation-related plasma protein levels and association with adiposity measurements in young adults
Source: Sci Rep. 2021 May 31;11:11391. doi: 10.1038/s41598-021-90843-x (PMC8166979; doi:10.1038/s41598-021-90843-x)
Supplement: Supplementary file 1 — Supplementary Tables. [file 41598_2021_90843_MOESM1_ESM.pdf]

SUPPLEMENT

Inflammation-related plasma protein levels and association with adiposity measurements in young adults

Klevebro S, Björkander S, Ekström S, Merid SK, Gruzieva O, Mälarstig A, Johansson Å, Kull I, Bergström A, Melén E

Table S1. Comparison of characteristics among the study population and participants in the BAMSE cohort not fulfilling the inclusion criteria

Table S2. Levels of the 71 inflammatory proteins included in the analyses by sex

Table S3. Association between body composition measurements and protein levels

Table S4. Association between %BF and protein levels stratified by three BMI categories

Table S5. Association between %BF and protein levels adjusted for physical activity

Table S6. Full and alternative protein names

Table S1. Comparison of characteristics among the study population and participants in the BAMSE cohort not fulfilling the inclusion criteria

	Included in study population *(n=2074)* *n (%)*	***Not included in study population*** *(n=2015)* *n (%)*	*P-value*[1]
Male sex (n=4089)	927 (44.7)	1138 (56.5)	<0.001
Parental professional worker at baseline (n=4018)	1740 (85.4)	1583 (79.9)	<0.001
Maternal smoking in pregnancy and/or infancy (n=4086)	255 (12.3)	308 (15.3)	<0.001
Any parent born outside of Scandinavia (n=3398)	281 (15.1)	262 (17.1)	0.11
Breastfeeding ≥4 months (n=3919)	1621 (80.3)	1495 (78.7)	0.21
High physical activity level at 16 years (n=2923)	1181 (67.3)	809 (69.3)	0.44
Overweight at 16 years (n=2599)	241 (14.3)	178 (19.6)	<0.001

[1] P-values estimated using the chi-2 test

[2] Levels of physical activity according to IPAQ (International Physical Activity Questionnaire) guidelines. High: ≥7hours/week of moderate to vigorous activity or ≥ 3.5 hours/week of vigorous activity

Table S2. *Levels of the 71 inflammatory proteins included in the analyses by sex*

Protein	***Females (n=1147)***		***Males (n=927)***		*p-value*
	Mean (sd)	*Median (p25 - p75)*	*Mean (sd)*	*Median (p25 - p75)*	
4EBP1	8.49 (0.85)	8.45 (7.92 - 9.00)	9.08 (0.87)	9.09 (8.59 - 9.65)	<0.001 [abc]
ADA	5.59 (0.41)	5.55 (5.32 - 5.80)	5.82 (0.42)	5.78 (5.55 - 6.05)	<0.001 [abc]
AXIN1	5.45 (0.89)	5.49 (4.97 - 5.98)	6.01 (0.93)	6.08 (5.60 - 6.58)	<0.001 [abc]
CASP8	1.72 (0.51)	1.66 (1.43 - 1.92)	2.18 (0.59)	2.11 (1.80 - 2.52)	<0.001 [abc]
CCL3	4.17 (0.51)	4.12 (3.86 - 4.40)	4.28 (0.44)	4.22 (3.98 - 4.53)	0.053 [a]
CCL4	5.50 (0.53)	5.47 (5.15 - 5.79)	5.74 (0.55)	5.67 (5.37 - 6.06)	0.011 [ab]
CCL11	6.19 (0.42)	6.18 (5.90 - 6.45)	6.40 (0.36)	6.37 (6.15 - 6.62)	0.141 [a]
CCL19	8.78 (0.67)	8.67 (8.35 - 9.07)	8.79 (0.55)	8.71 (8.43 - 9.07)	<0.001 [abc]
CCL20	6.38 (0.81)	6.28 (5.86 - 6.75)	6.46 (0.81)	6.34 (5.98 - 6.75)	<0.001 [abc]
CCL23	10.06 (0.46)	10.06 (9.77 - 10.36)	10.03 (0.44)	10.03 (9.76 - 10.29)	<0.001 [abc]
CCL25	5.70 (0.50)	5.71 (5.35 - 6.01)	5.82 (0.47)	5.79 (5.50 - 6.11)	<0.001 [abc]
CCL28	2.06 (0.38)	2.02 (1.82 - 2.25)	1.91 (0.35)	1.88 (1.70 - 2.08)	<0.001 [abc]
CD5	5.16 (0.46)	5.09 (4.88 - 5.31)	5.21 (0.46)	5.11 (4.92 - 5.36)	<0.001 [abc]
CD6	5.46 (0.81)	5.33 (4.96 - 5.68)	5.60 (0.72)	5.45 (5.16 - 5.86)	0.011 [ab]
CD8A	10.58 (0.57)	10.55 (10.20 - 10.96)	10.57 (0.57)	10.56 (10.19 - 10.93)	<0.001 [abc]
CD40	11.72 (0.43)	11.70 (11.47 - 11.97)	11.91 (0.44)	11.91 (11.66 - 12.18)	0.721
CD244	7.20 (0.37)	7.18 (6.97 - 7.40)	7.37 (0.38)	7.35 (7.12 - 7.59)	0.543 [abc]
CDCP1	2.16 (0.40)	2.11 (1.91 - 2.34)	2.14 (0.38)	2.10 (1.91 - 2.33)	<0.001 [abc]
CSF1	9.86 (0.24)	9.83 (9.69 - 10.02)	9.75 (0.18)	9.75 (9.63 - 9.86)	<0.001 [bc]
CST5	4.94 (0.42)	4.92 (4.66 - 5.19)	5.01 (0.42)	4.96 (4.69 - 5.27)	0.669 [a]
CX3CL1	5.71 (0.33)	5.71 (5.48 - 5.93)	5.71 (0.33)	5.70 (5.47 - 5.93)	0.002 [abc]
CXCL1	9.25 (0.75)	9.35 (8.76 - 9.74)	9.35 (0.76)	9.43 (8.92 - 9.89)	0.472 [a]
CXCL5	11.31 (0.98)	11.36 (10.69 - 11.99)	11.29 (0.95)	11.33 (10.75 - 11.97)	0.086 [c]
CXCL6	8.78 (0.72)	8.76 (8.31 - 9.19)	9.14 (0.73)	9.10 (8.63 - 9.61)	0.539 [a]
CXCL9	6.57 (0.78)	6.43 (6.08 - 6.85)	6.57 (0.73)	6.45 (6.13 - 6.84)	<0.001 [abc]

CXCL10	8.91 (0.84)	8.76 (8.40 - 9.18)	8.92 (0.79)	8.77 (8.41 - 9.20)	0.548
CXCL11	8.19 (0.83)	8.07 (7.63 - 8.66)	8.12 (0.81)	8.04 (7.56 - 8.56)	<0.001 [abc]
DNER	8.96 (0.21)	8.97 (8.82 - 9.11)	9.08 (0.20)	9.08 (8.95 - 9.22)	<0.001 [abc]
ENRAGE	1.84 (0.59)	1.77 (1.48 - 2.08)	2.22 (0.55)	2.16 (1.85 - 2.52)	<0.001 [abc]
FGF19	7.94 (0.96)	7.90 (7.27 - 8.60)	7.92 (0.90)	7.89 (7.31 - 8.48)	0.708 [a]
FGF21	3.78 (1.30)	3.68 (2.90 - 4.59)	3.71 (1.25)	3.68 (2.87 - 4.48)	0.348 [a c]
FGF23	2.56 (0.53)	2.46 (2.24 - 2.74)	2.46 (0.35)	2.44 (2.24 - 2.63)	0.003 [bc]
Flt3L	8.98 (0.38)	8.97 (8.74 - 9.19)	8.80 (0.36)	8.82 (8.57 - 9.03)	<0.001 [abc]
GDNF	2.24 (0.43)	2.22 (1.96 - 2.46)	2.29 (0.31)	2.28 (2.09 - 2.48)	<0.001 [bc]
HGF	8.23 (0.41)	8.20 (7.95 - 8.47)	8.48 (0.37)	8.43 (8.22 - 8.69)	<0.001 [abc]
IFNgamma	6.09 (1.09)	5.86 (5.45 - 6.43)	6.04 (1.08)	5.83 (5.40 - 6.36)	0.176
IL6	2.61 (0.60)	2.50 (2.20 - 2.89)	2.60 (0.63)	2.48 (2.18 - 2.88)	0.002 [abc]
IL7	3.45 (0.61)	3.40 (3.06 - 3.76)	3.54 (0.60)	3.48 (3.18 - 3.86)	0.116 [a]
IL8	4.46 (0.50)	4.42 (4.14 - 4.71)	4.64 (0.50)	4.57 (4.32 - 4.87)	<0.001 [bc]
IL10	3.90 (0.69)	3.81 (3.56 - 4.09)	3.95 (0.57)	3.87 (3.63 - 4.13)	<0.001 [abc]
IL10RB	5.68 (0.25)	5.68 (5.52 - 5.84)	5.70 (0.26)	5.69 (5.53 - 5.86)	0.241 [a]
IL12B	5.80 (0.56)	5.80 (5.42 - 6.16)	5.65 (0.52)	5.65 (5.31 - 6.00)	<0.001 [abc]
IL15RA	1.34 (0.25)	1.33 (1.18 - 1.48)	1.45 (0.23)	1.44 (1.31 - 1.59)	<0.001 [ab]
IL17A	1.85 (0.53)	1.77 (1.51 - 2.09)	1.87 (0.53)	1.79 (1.53 - 2.08)	0.499 [abc]
IL18	7.98 (0.50)	7.95 (7.66 - 8.30)	8.11 (0.51)	8.08 (7.81 - 8.41)	<0.001 [abc]
IL18R1	8.13 (0.39)	8.13 (7.86 - 8.38)	8.21 (0.38)	8.21 (7.94 - 8.46)	<0.001 [abc]
LAPTGFbeta1	7.40 (0.38)	7.36 (7.15 - 7.60)	7.51 (0.41)	7.48 (7.25 - 7.71)	<0.001 [abc]
LIFR	3.65 (0.24)	3.66 (3.49 - 3.80)	3.67 (0.19)	3.66 (3.54 - 3.78)	0.042 [abc]
MCP1	10.88 (0.43)	10.83 (10.61 - 11.08)	11.04 (0.39)	10.98 (10.79 - 11.21)	<0.001 [abc]
MCP2	8.22 (0.66)	8.25 (7.87 - 8.57)	8.41 (0.67)	8.46 (8.03 - 8.78)	<0.001 [abc]
MCP4	12.76 (0.67)	12.71 (12.33 - 13.16)	13.03 (0.66)	12.97 (12.59 - 13.41)	<0.001 [abc]
MMP1	9.09 (1.05)	9.03 (8.43 - 9.68)	9.33 (0.96)	9.28 (8.70 - 9.88)	<0.001 [abc]
MMP10	8.13 (0.67)	8.05 (7.70 - 8.45)	8.14 (0.58)	8.08 (7.77 - 8.50)	0.358

NT3	2.92 (0.42)	2.87 (2.69 - 3.05)	2.95 (0.39)	2.90 (2.71 - 3.11)	0.005 [bc]
OPG	10.05 (0.37)	10.01 (9.81 - 10.26)	9.91 (0.27)	9.92 (9.73 - 10.09)	<0.001 [abc]
OSM	3.61 (1.02)	3.47 (2.84 - 4.30)	3.85 (0.95)	3.74 (3.15 - 4.48)	<0.001 [abc]
PDL1	6.54 (0.39)	6.50 (6.31 - 6.69)	6.74 (0.38)	6.69 (6.51 - 6.91)	<0.001 [abc]
SCF	8.96 (0.40)	9.02 (8.76 - 9.24)	9.11 (0.32)	9.13 (8.96 - 9.33)	<0.001 [abc]
SIRT2	6.00 (1.03)	6.05 (5.40 - 6.67)	6.47 (1.07)	6.54 (5.96 - 7.16)	<0.001 [abc]
ST1A1	3.53 (0.70)	3.55 (3.10 - 3.96)	3.92 (0.71)	3.94 (3.52 - 4.41)	<0.001 [abc]
STAMBP	6.26 (0.90)	6.23 (5.73 - 6.79)	6.85 (0.96)	6.85 (6.36 - 7.45)	<0.001 [abc]
TGFalpha	3.11 (0.37)	3.05 (2.90 - 3.21)	3.15 (0.36)	3.07 (2.92 - 3.25)	0.016 [bc]
TNF	3.05 (0.71)	2.88 (2.65 - 3.21)	3.10 (0.72)	2.96 (2.73 - 3.24)	0.002 [abc]
TNFB	4.96 (0.43)	4.96 (4.73 - 5.22)	4.87 (0.42)	4.90 (4.65 - 5.09)	<0.001 [abc]
TNFRSF9	6.70 (0.35)	6.70 (6.46 - 6.91)	6.84 (0.31)	6.82 (6.62 - 7.05)	<0.001 [abc]
TNFSF14	4.00 (0.59)	3.92 (3.61 - 4.31)	4.27 (0.64)	4.17 (3.85 - 4.61)	<0.001 [abc]
TRAIL	7.75 (0.36)	7.77 (7.52 - 7.98)	8.01 (0.28)	8.00 (7.83 - 8.18)	<0.001 [abc]
TRANCE	4.56 (0.67)	4.60 (4.10 - 5.03)	4.92 (0.57)	4.94 (4.56 - 5.29)	<0.001 [abc]
TWEAK	9.61 (0.37)	9.64 (9.38 - 9.86)	9.83 (0.28)	9.82 (9.64 - 10.00)	<0.001 [abc]
uPA	10.11 (0.35)	10.13 (9.88 - 10.36)	10.24 (0.28)	10.24 (10.09 - 10.41)	0.294 [a]
VEGFA	10.24 (0.32)	10.22 (10.03 - 10.43)	10.26 (0.32)	10.22 (10.05 - 10.44)	<0.001 [abc]

Protein levels in NPX unit.

Linear regression model using normalized protein values (rank-based inverse normal transformation).

p-value at 0.05 FDR

[a] p-value <0.05 adjusted for percentage body fat

[b] p-value <0.05 adjusted for body mass index

[c] p-value <0.05 adjusted for visceral fat rating

sd, standard deviation; p25, 25th percentile; p75, 75th percentile

Table S3. Association between body composition measurements and protein levels

	% Body Fat			Body Mass Index			Visceral Fat Rating			Waist Circumference		
Protein	*Coef*	*95% CI*	*p-value*	*Coef*	*95% CI*	*p-value*	*Coef*	*95% CI*	*p-value*	*Coef*	*95% CI*	*p-value*
4EBP1	0.19	0.13 - 0.25	<0.001	0.02	0.01 - 0.03	<0.001	0.05	0.03 - 0.07	<0.001	0.05	0.03 - 0.07	<0.001
ADA	0.17	0.10 - 0.23	<0.001	0.04	0.03 - 0.05	<0.001	0.04	0.03 - 0.06	<0.001	0.07	0.05 - 0.09	<0.001
AXIN1	0.20	0.14 - 0.27	<0.001	0.03	0.02 - 0.04	<0.001	0.05	0.03 - 0.07	<0.001	0.06	0.04 - 0.08	<0.001
CASP8	0.08	0.02 - 0.14	0.019	0.01	0.00 - 0.02	0.009	0.02	0.00 - 0.03	0.050	0.02	0.00 - 0.04	0.035
CCL3	0.42	0.36 - 0.49	<0.001	0.06	0.05 - 0.07	<0.001	0.11	0.09 - 0.13	<0.001	0.12	0.10 - 0.14	<0.001
CCL4	0.28	0.21 - 0.34	<0.001	0.04	0.03 - 0.05	<0.001	0.08	0.07 - 0.10	<0.001	0.09	0.07 - 0.11	<0.001
CCL11	-0.16	-0.22 - -0.09	<0.001	-0.02	-0.03 - -0.01	<0.001	-0.03	-0.05 - -0.01	<0.001	-0.02	-0.05 - 0.00	0.046
CCL19	0.25	0.19 - 0.32	<0.001	0.04	0.03 - 0.05	<0.001	0.07	0.05 - 0.08	<0.001	0.09	0.07 - 0.11	<0.001
CCL20	0.12	0.06 - 0.19	0.001	0.02	0.01 - 0.03	0.004	0.04	0.02 - 0.06	<0.001	0.05	0.03 - 0.07	<0.001
CCL23	-0.09	-0.16 - -0.02	0.010	-0.02	-0.03 - -0.01	0.004	-0.02	-0.04 - 0.00	0.023	-0.03	-0.05 - 0.00	0.035
CCL25	-0.07	-0.14 - -0.01	0.039	-0.01	-0.02 - 0.00	0.034	-0.01	-0.03 - 0.00	0.153	-0.01	-0.04 - 0.01	0.277
CCL28	-0.16	-0.22 - -0.09	<0.001	-0.03	-0.04 - -0.02	<0.001	-0.04	-0.06 - -0.03	<0.001	-0.06	-0.08 - -0.03	<0.001
CD5	0.14	0.07 - 0.21	<0.001	0.03	0.02 - 0.04	<0.001	0.04	0.03 - 0.06	<0.001	0.06	0.04 - 0.08	<0.001
CD6	0.03	-0.04 - 0.09	0.449	0.01	0.00 - 0.02	0.055	0.01	0.00 - 0.03	0.090	0.03	0.01 - 0.05	0.013
CD8A	0.04	-0.03 - 0.11	0.262	0.00	-0.01 - 0.01	0.958	0.01	-0.01 - 0.02	0.532	0.01	-0.01 - 0.04	0.294
CD40	0.21	0.14 - 0.27	<0.001	0.03	0.02 - 0.04	<0.001	0.05	0.03 - 0.07	<0.001	0.07	0.05 - 0.09	<0.001
CD244	0.10	0.04 - 0.17	0.004	0.01	0.00 - 0.03	0.013	0.02	0.00 - 0.04	0.021	0.03	0.01 - 0.06	0.005
CDCP1	0.49	0.42 - 0.56	<0.001	0.08	0.07 - 0.09	<0.001	0.15	0.13 - 0.17	<0.001	0.18	0.15 - 0.20	<0.001
CSF1	0.25	0.19 - 0.32	<0.001	0.04	0.03 - 0.05	<0.001	0.07	0.05 - 0.09	<0.001	0.08	0.05 - 0.10	<0.001
CST5	-0.15	-0.21 - -0.08	<0.001	-0.03	-0.05 - -0.02	<0.001	-0.03	-0.05 - -0.02	<0.001	-0.06	-0.08 - -0.03	<0.001
CX3CL1	-0.11	-0.17 - -0.04	0.003	-0.02	-0.03 - -0.01	<0.001	-0.03	-0.05 - -0.01	0.005	-0.05	-0.07 - -0.02	<0.001
CXCL1	0.13	0.07 - 0.20	<0.001	0.02	0.01 - 0.03	0.002	0.03	0.01 - 0.05	0.001	0.04	0.01 - 0.06	0.002
CXCL5	0.17	0.11 - 0.24	<0.001	0.02	0.01 - 0.03	<0.001	0.04	0.02 - 0.06	<0.001	0.05	0.03 - 0.07	<0.001

CXCL6	0.13	0.06	-	0.20	<0.001	0.02	0.01	-	0.03	0.002	0.03	0.01	-	0.05	0.001	0.03	0.01	-	0.06	0.003
CXCL9	0.04	-0.02	-	0.11	0.229	0.00	-0.01	-	0.02	0.459	0.00	-0.02	-	0.02	0.877	0.02	0.00	-	0.05	0.046
CXCL10	0.15	0.08	-	0.21	<0.001	0.03	0.02	-	0.04	<0.001	0.04	0.02	-	0.05	<0.001	0.05	0.03	-	0.07	<0.001
CXCL11	0.05	-0.02	-	0.12	0.158	0.00	-0.01	-	0.02	0.441	0.01	0.00	-	0.03	0.182	0.01	-0.01	-	0.04	0.221
DNER	-0.19	-0.26	-	-0.13	<0.001	-0.02	-0.03	-	-0.01	0.001	-0.04	-0.06	-	-0.02	<0.001	-0.06	-0.08	-	-0.04	<0.001
ENRAGE	0.09	0.02	-	0.15	0.010	0.02	0.01	-	0.03	<0.001	0.03	0.02	-	0.05	<0.001	0.04	0.01	-	0.06	0.001
FGF19	-0.11	-0.18	-	-0.04	0.002	-0.02	-0.03	-	-0.01	<0.001	-0.03	-0.05	-	-0.01	0.014	-0.03	-0.06	-	-0.01	0.015
FGF21	0.33	0.27	-	0.40	<0.001	0.04	0.02	-	0.05	<0.001	0.09	0.07	-	0.10	<0.001	0.09	0.06	-	0.11	<0.001
FGF23	0.23	0.16	-	0.30	<0.001	0.03	0.02	-	0.04	<0.001	0.07	0.05	-	0.09	<0.001	0.08	0.06	-	0.10	<0.001
Flt3L	-0.03	-0.10	-	0.04	0.436	0.01	-0.01	-	0.02	0.421	0.01	-0.01	-	0.03	0.306	0.01	-0.01	-	0.03	0.429
GDNF	-0.12	-0.19	-	-0.06	0.001	-0.01	-0.02	-	0.00	0.036	-0.01	-0.02	-	0.01	0.549	-0.03	-0.05	-	-0.01	0.014
HGF	0.34	0.27	-	0.40	<0.001	0.06	0.05	-	0.07	<0.001	0.11	0.09	-	0.12	<0.001	0.12	0.10	-	0.14	<0.001
IFNgamma	0.09	0.03	-	0.16	0.009	0.02	0.01	-	0.03	0.001	0.02	0.00	-	0.03	0.029	0.04	0.02	-	0.06	<0.001
IL6	0.60	0.54	-	0.66	<0.001	0.08	0.07	-	0.09	<0.001	0.14	0.12	-	0.17	<0.001	0.16	0.14	-	0.18	<0.001
IL7	0.20	0.14	-	0.27	<0.001	0.03	0.02	-	0.04	<0.001	0.05	0.04	-	0.07	<0.001	0.07	0.04	-	0.09	<0.001
IL8	0.13	0.06	-	0.20	<0.001	0.02	0.01	-	0.03	0.005	0.04	0.02	-	0.06	<0.001	0.05	0.02	-	0.07	<0.001
IL10	0.12	0.05	-	0.18	0.001	0.02	0.01	-	0.03	0.003	0.03	0.01	-	0.05	0.002	0.05	0.02	-	0.07	<0.001
IL10RB	0.24	0.17	-	0.31	<0.001	0.04	0.03	-	0.05	<0.001	0.07	0.05	-	0.09	<0.001	0.08	0.06	-	0.11	<0.001
IL12B	0.22	0.16	-	0.29	<0.001	0.03	0.02	-	0.05	<0.001	0.05	0.03	-	0.07	<0.001	0.08	0.06	-	0.10	<0.001
IL15RA	0.08	0.02	-	0.15	0.018	0.02	0.01	-	0.03	0.003	0.02	0.01	-	0.04	0.006	0.04	0.02	-	0.06	0.001
IL17A	0.06	-0.01	-	0.13	0.114	0.01	0.00	-	0.02	0.255	0.01	-0.01	-	0.03	0.190	0.03	0.01	-	0.06	0.005
IL18	0.28	0.21	-	0.34	<0.001	0.05	0.04	-	0.06	<0.001	0.08	0.06	-	0.10	<0.001	0.10	0.08	-	0.13	<0.001
IL18R1	0.49	0.42	-	0.56	<0.001	0.07	0.06	-	0.09	<0.001	0.14	0.11	-	0.16	<0.001	0.15	0.13	-	0.17	<0.001
LAPTGFbeta1	0.13	0.07	-	0.20	<0.001	0.03	0.02	-	0.04	<0.001	0.05	0.03	-	0.07	<0.001	0.06	0.03	-	0.08	<0.001
LIFR	-0.29	-0.35	-	-0.22	<0.001	-0.03	-0.04	-	-0.02	<0.001	-0.05	-0.07	-	-0.03	<0.001	-0.06	-0.08	-	-0.03	<0.001
MCP1	0.13	0.06	-	0.19	0.001	0.03	0.02	-	0.04	<0.001	0.05	0.03	-	0.07	<0.001	0.06	0.04	-	0.08	<0.001
MCP2	0.07	0.01	-	0.14	0.042	0.01	0.00	-	0.02	0.180	0.02	0.00	-	0.04	0.063	0.02	-0.01	-	0.04	0.146
MCP4	0.10	0.03	-	0.16	0.009	0.02	0.01	-	0.03	<0.001	0.05	0.03	-	0.06	<0.001	0.05	0.03	-	0.07	<0.001

MMP1	-0.03	-0.10 - 0.04	0.407	0.00	-0.01 - 0.01	0.894	0.01	-0.01 - 0.03	0.253	0.00	-0.02 - 0.03	0.691
MMP10	-0.06	-0.12 - 0.01	0.131	-0.01	-0.03 - 0.00	0.014	-0.02	-0.04 - 0.00	0.032	-0.01	-0.04 - 0.01	0.263
NT3	-0.07	-0.14 - 0.00	0.064	-0.01	-0.02 - 0.01	0.378	-0.01	-0.03 - 0.01	0.217	0.00	-0.03 - 0.02	0.796
OPG	-0.04	-0.11 - 0.02	0.219	0.00	-0.01 - 0.01	0.890	-0.01	-0.02 - 0.01	0.532	-0.01	-0.03 - 0.01	0.429
OSM	0.19	0.12 - 0.26	<0.001	0.03	0.02 - 0.04	<0.001	0.06	0.04 - 0.08	<0.001	0.07	0.04 - 0.09	<0.001
PDL1	0.12	0.06 - 0.19	0.001	0.02	0.01 - 0.03	<0.001	0.03	0.01 - 0.05	0.001	0.04	0.01 - 0.06	0.002
SCF	-0.19	-0.26 - -0.11	<0.001	-0.03	-0.04 - -0.02	<0.001	-0.06	-0.08 - -0.04	<0.001	-0.05	-0.08 - -0.03	<0.001
SIRT2	0.19	0.13 - 0.26	<0.001	0.03	0.02 - 0.04	<0.001	0.05	0.03 - 0.07	<0.001	0.05	0.03 - 0.08	<0.001
ST1A1	0.11	0.04 - 0.17	0.002	0.01	0.00 - 0.02	0.019	0.02	0.00 - 0.04	0.050	0.03	0.00 - 0.05	0.024
STAMBP	0.18	0.12 - 0.25	<0.001	0.03	0.02 - 0.04	<0.001	0.05	0.03 - 0.06	<0.001	0.06	0.04 - 0.08	<0.001
TGFalpha	-0.04	-0.11 - 0.03	0.253	-0.01	-0.02 - 0.00	0.184	0.00	-0.01 - 0.02	0.685	-0.01	-0.03 - 0.02	0.655
TNF	0.14	0.07 - 0.21	<0.001	0.02	0.01 - 0.03	0.002	0.03	0.01 - 0.05	0.001	0.05	0.03 - 0.07	<0.001
TNFB	-0.01	-0.08 - 0.05	0.716	0.00	-0.01 - 0.01	0.674	-0.01	-0.03 - 0.01	0.306	0.01	-0.01 - 0.03	0.385
TNFRSF9	0.08	0.01 - 0.15	0.030	0.02	0.01 - 0.03	0.001	0.03	0.01 - 0.04	0.008	0.05	0.03 - 0.07	<0.001
TNFSF14	0.27	0.21 - 0.34	<0.001	0.04	0.03 - 0.05	<0.001	0.08	0.06 - 0.09	<0.001	0.09	0.07 - 0.11	<0.001
TRAIL	0.24	0.18 - 0.30	<0.001	0.05	0.04 - 0.06	<0.001	0.07	0.05 - 0.09	<0.001	0.09	0.07 - 0.11	<0.001
TRANCE	0.29	0.23 - 0.35	<0.001	0.04	0.03 - 0.06	<0.001	0.07	0.05 - 0.09	<0.001	0.11	0.08 - 0.13	<0.001
TWEAK	-0.09	-0.16 - -0.03	0.007	-0.02	-0.03 - -0.01	0.005	-0.02	-0.04 - -0.01	0.010	-0.02	-0.05 - 0.00	0.043
uPA	-0.06	-0.13 - 0.01	0.115	0.01	0.00 - 0.02	0.274	-0.01	-0.02 - 0.01	0.613	0.01	-0.01 - 0.04	0.244
VEGFA	0.35	0.29 - 0.42	<0.001	0.06	0.05 - 0.07	<0.001	0.10	0.07 - 0.12	<0.001	0.11	0.09 - 0.14	<0.001

Linear regression model using normalized protein values (rank-based inverse normal transformation). Adjusted for sex, smoking, e-cigarette use, snuff use, and age at sampling.
Coefficients per 10% increase in %BF and 5cm increase in waist circumference.
p-value at 0.05 FDR.

Table S4. Association between %BF and protein levels stratified by three BMI categories

Protein	***Normal weight (18.5 to <25 kg/ m^2)*** *Coef (95% CI)*	***Overweight (≥25 to <30 kg/ m^2)*** *Coef (95% CI)*	***Obesity (≥30 kg/ m^2)*** *Coef (95% CI)*
CCL3	0.31 (0.21 - 0.51)	0.61 (0.31 - 0.91)	0.61 (0.21 - 1.01)
CCL20	0.11 (0.01 - 0.21)	0.31 (0.11 - 0.61)	0.61 (0.21 - 1.11)
CDCP1	0.31 (0.21 - 0.41)	0.71 (0.41 - 1.01)	0.71 (0.31 - 1.21)
DNER	-0.21 (-0.31 - -0.11)	-0.41 (-0.61 - -0.11)	-0.71 (-1.31 - -0.21)
FGF21	0.31 (0.21 - 0.41)	1.01 (0.71 - 1.21)	0.61 (0.11 - 1.11)
FGF23	0.11 (-0.11 - 0.21)	0.51 (0.21 - 0.81)	0.51 (0.11 - 0.91)
HGF	0.11 (0.01 - 0.21)	0.51 (0.31 - 0.71)	0.61 (0.31 - 1.01)
IL6	0.51 (0.41 - 0.61)	0.81 (0.61 - 1.01)	0.61 (0.31 - 0.91)
IL18	0.11 (0.01 - 0.31)	0.31 (0.11 - 0.61)	0.51 (0.01 - 1.01)
LAPTGFbeta1	0.01 (-0.11 - 0.11)	0.11 (-0.21 - 0.41)	0.31 (-0.11 - 0.61)
MCP1	-0.11 (-0.21 - 0.01)	0.11 (-0.11 - 0.41)	0.51 (0.11 - 0.81)
MCP4	-0.11 (-0.21 - 0.01)	0.01 (-0.31 - 0.21)	0.21 (-0.21 - 0.71)
OSM	0.01 (-0.11 - 0.11)	0.01 (-0.31 - 0.31)	0.31 (0.01 - 0.71)
TNFSF14	0.21 (0.01 - 0.31)	0.21 (-0.11 - 0.41)	0.31 (-0.11 - 0.61)

Table S5. Association between %BF and protein levels adjusted for physical activity

Protein	*Coef*	*95% CI*			*p-value*
4EBP1	0.18	0.11	-	0.25	<0.001
ADA	0.17	0.10	-	0.24	<0.001
AXIN1	0.20	0.13	-	0.27	<0.001
CASP8	0.08	0.01	-	0.15	0.042
CCL3	0.42	0.35	-	0.49	<0.001
CCL4	0.26	0.19	-	0.34	<0.001
CCL11	-0.16	-0.23	-	-0.08	<0.001
CCL19	0.24	0.16	-	0.31	<0.001
CCL20	0.10	0.03	-	0.18	0.012
CCL23	-0.08	-0.15	-	0.00	0.063
CCL25	-0.10	-0.18	-	-0.03	0.009
CCL28	-0.14	-0.21	-	-0.06	0.001
CD5	0.13	0.05	-	0.21	0.002
CD6	0.02	-0.06	-	0.09	0.662
CD8A	0.04	-0.04	-	0.11	0.388
CD40	0.21	0.13	-	0.28	<0.001
CD244	0.11	0.03	-	0.19	0.007
CDCP1	0.46	0.38	-	0.54	<0.001
CSF1	0.24	0.17	-	0.31	<0.001
CST5	-0.15	-0.23	-	-0.08	<0.001
CX3CL1	-0.15	-0.22	-	-0.07	<0.001
CXCL1	0.15	0.08	-	0.23	<0.001
CXCL5	0.18	0.10	-	0.25	<0.001

CXCL6	0.13	0.05	-	0.20	0.002
CXCL9	0.04	-0.03	-	0.11	0.311
CXCL10	0.13	0.06	-	0.21	0.001
CXCL11	0.05	-0.02	-	0.13	0.179
DNER	-0.22	-0.30	-	-0.15	<0.001
ENRAGE	0.09	0.02	-	0.16	0.020
FGF19	-0.11	-0.19	-	-0.04	0.005
FGF21	0.32	0.25	-	0.39	<0.001
FGF23	0.22	0.15	-	0.30	<0.001
Flt3L	-0.03	-0.11	-	0.05	0.442
GDNF	-0.15	-0.22	-	-0.07	<0.001
HGF	0.33	0.26	-	0.40	<0.001
IFNgamma	0.11	0.04	-	0.18	0.005
IL6	0.59	0.53	-	0.66	<0.001
IL7	0.21	0.14	-	0.29	<0.001
IL8	0.12	0.05	-	0.20	0.003
IL10	0.09	0.01	-	0.16	0.030
IL10RB	0.23	0.15	-	0.30	<0.001
IL12B	0.22	0.14	-	0.29	<0.001
IL15RA	0.07	0.00	-	0.15	0.067
IL17A	0.10	0.02	-	0.18	0.019
IL18	0.28	0.21	-	0.35	<0.001
IL18R1	0.47	0.39	-	0.54	<0.001
LAPTGFbeta1	0.13	0.05	-	0.20	0.002
LIFR	-0.31	-0.38	-	-0.24	<0.001
MCP1	0.10	0.03	-	0.17	0.012
MCP2	0.07	-0.01	-	0.15	0.082
MCP4	0.08	0.00	-	0.16	0.063

MMP1	-0.03	-0.11	-	0.04	0.424
MMP10	-0.06	-0.14	-	0.02	0.169
NT3	-0.06	-0.14	-	0.02	0.153
OPG	-0.06	-0.13	-	0.02	0.146
OSM	0.21	0.13	-	0.29	<0.001
PDL1	0.12	0.05	-	0.20	0.002
SCF	-0.19	-0.27	-	-0.11	<0.001
SIRT2	0.19	0.12	-	0.27	<0.001
ST1A1	0.11	0.04	-	0.19	0.006
STAMBP	0.18	0.11	-	0.25	<0.001
TGFalpha	-0.05	-0.13	-	0.03	0.226
TNF	0.15	0.08	-	0.23	<0.001
TNFB	-0.02	-0.09	-	0.06	0.652
TNFRSF9	0.07	0.00	-	0.15	0.066
TNFSF14	0.27	0.20	-	0.35	<0.001
TRAIL	0.23	0.16	-	0.30	<0.001
TRANCE	0.28	0.21	-	0.35	<0.001
TWEAK	-0.09	-0.16	-	-0.02	0.020
uPA	-0.08	-0.16	-	-0.01	0.039
VEGFA	0.35	0.28	-	0.42	<0.001

Linear regression model using normalized protein values (rank-based inverse normal transformation). Adjusted for sex, smoking, e-cigarette use, snuff use, age at sampling, and level of physical activity.

Coefficients per 10% increase in %BF.

p-value at 0.05 FDR.

n=1728

Table S6. Full and alternative protein names

Protein	*Full name in Olink inflammation panel*	*Alternative names*
4EBP1	Eukaryotic translation initiation factor 4E-binding protein 1	
ADA	Adenosine Deaminase	
AXIN1	Axin-1	
CASP8	Caspase-8	
CCL3	C-C motif chemokine 3	MIP-1 alpha
CCL4	C-C motif chemokine 4	MIP-1 beta
CCL11	C-C motif chemokine 11	Eotaxin-1, eosinophil chemotactic protein
CCL19	C-C motif chemokine 19	MIP-3 beta
CCL20	C-C motif chemokine 20	MIP-3 alpha, LARC
CCL23	C-C motif chemokine 23	MIP-3
CCL25	C-C motif chemokine 25	TECK
CCL28	C-C motif chemokine 28	MEC
CD5	Cluster of differentiation 5	
CD6	Cluster of differentiation 6	
CD8A	Cluster of differentiation 8a	
CD40	Cluster of differentiation 40	TNFRSF5
CD244	Natural Killer cell receptor 2B4	SLAMF4, CD244
CDCP1	CUB domain-containing protein 1	CD318, TRASK
CSF1	Macrophage colony-stimulating factor 1	M-CFS
CST5	Cystatin D	
CX3CL1	Fractalkine	
CXCL1	C-X-C motif chemokine 1	Gro 1, Gro alpha
CXCL5	C-X-C motif chemokine 5	ENA-78
CXCL6	C-X-C motif chemokine 6	GCP-2
CXCL9	C-X-C motif chemokine 9	MIG
CXCL10	C-X-C motif chemokine 10	IP-10

CXCL11	C-X-C motif chemokine 11	I-TAC
DNER	Delta and Notch-like epidermal growth factor-related receptor	
ENRAGE	Protein S100-A12	
FGF19	Fibroblast growth factor 19	
FGF21	Fibroblast growth factor 21	
FGF23	Fibroblast growth factor 23	
Flt3L	Fms-related tyrosine kinase 3 ligand	FLT3LG
GDNF	Glial cell line-derived neurotrophic factor	
HGF	Hepatocyte growth factor	
IFNgamma	Interferon-gamma	
IL6	Interleukin-6	
IL7	Interleukin-7	
IL8	Interleukin-8	CXCL8, Neutrophil chemotactic factor
IL10	Interleukin-10	
IL10RB	Interleukin-10 receptor beta	
IL12B	Interleukin-12 subunit beta	
IL15RA	Interleukin-15 receptor alpha	
IL17A	Interleukin-17A	
IL18	Interleukin-18	
IL18R1	Interleukin-18 receptor 1	
LAPTGFbeta1	Latency-associated peptide transforming growth factor beta-1	
LIFR	Leukemia inhibitory factor receptor	CD118
MCP1	Monocyte chemotactic protein 1	CCL2
MCP2	Monocyte chemotactic protein 2	CCL8
MCP4	Monocyte chemotactic protein 4	CCL13
MMP1	Matrix metalloproteinase-1	
MMP10	Matrix metalloproteinase-10	

NT3	Neurotrophin-3	
OPG	Osteoprotegerin	OCIF, TNFRSF11B
OSM	Oncostatin-M	
PDL1	Programmed cell death ligand 1	CD274, B7-H1
SCF	Stem Cell Factor	KIT-ligand
SIRT2	SIR2-like protein 2	Sirtuin 2
ST1A1	Sulfotransferase 1A1	SULT1A1
STAMBP	STAM-binding protein	
TGFalpha	Transforming Growth Factor alpha	
TNF	Tumor necrosis factor	TNF alpha
TNFB	Tumor necrosis factor beta	Lymphotoxin alpha
TNFRSF9	Tumor necrosis factor receptor superfamily member 9	CD137, 4-1BB
TNFSF14	Tumor necrosis factor ligand superfamily member 14	CD258, LIGHT
TRAIL	TNF-related apoptosis-inducing ligand	CD253, TNFSF10
TRANCE	TNF-related activation-induced cytokine	CD254, TNFSF11, RANKL, OPGL, ODF
TWEAK	TNF-related weak inducer of apoptosis	TNFSF12, Apo3L
uPA	Urokinase-type plasminogen activator	
VEGFA	Vascular endothelial growth factor A	

CD: Cluster of Differentiation
